# Optical coherence tomography angiography of peripapillary vessel density in non-arteritic anterior ischemic optic neuropathy and demyelinating optic neuritis

**DOI:** 10.3389/fneur.2024.1432753

**Published:** 2024-10-29

**Authors:** Qing Xiao, Chuan-bin Sun, Zhiqiong Ma

**Affiliations:** ^1^Eye Center, Second Affiliated Hospital of Zhejiang University School of Medicine, Hangzhou, Zhejiang, China; ^2^Department of Ophthalmology, Affiliated Xiaoshan Hospital, Hangzhou Normal University, Hangzhou, Zhejiang, China

**Keywords:** optical coherence tomography angiography, vessel density, nonarteritic anterior ischemic optic neuropathy, optic neuritis, biomarker

## Abstract

**Background:**

In cases of optic disc edema or a pale optic disc, distinguishing an episode of optic neuritis (ON) from that of non-arteritic anterior ischemic optic neuropathy (NAION) during a clinical examination is challenging. Optical coherence tomography angiography (OCTA) can reveal differences in peripapillary vascular network structures and provide biomarkers for differential diagnosis.

**Methods:**

A total of 23 eyes with NAION, 22 eyes with demyelinating ON (DON), and 27 eyes from healthy participants were imaged using OCTA to observe the radial peripapillary capillaries (RPCs). Optical coherence tomography was used to measure peripapillary retinal nerve fiber layer (RNFL) thickness and the macular ganglion cell complex (mGCC). Data for all patients were recorded at 2–3 weeks and more than 3 months after the symptom onset.

**Results:**

A total of 23 affected eyes from 23 patients with NAION (average age 52.17 ± 7.92 years), 22 eyes from 22 patients with demyelinating optic neuritis (DON) (average age 47.88 ± 19.24 years), and 27 eyes from 27 healthy individuals (average age 46.43 ± 14.08 years) were included in the study. There were no significant differences in sex, age, and eye laterality between any two groups (*F* = 0.968, 0.475, 0.870; *p* > 0.05). Throughout the course of NAION and DON, the superior RPC, superior mGCC, and peripapillary RNFL decreased with time (*p* < 0.05). In contrast, the inferotemporal RPC and inferior mGCC did not decrease from the acute to chronic stage in NAION (*t* = 1.639, 0.834, *p* = 0.117, 0.413). Compared with the normal group, patients with NAION and DON exhibited a sharp reduction in the average RPC, RNFL, and GCC from the acute to the chronic stage (*p* < 0.05). Patients with DON exhibited a significant decrease in the inferotemporal RPC and inferior mGCC compared with the patients with NAION (*p* < 0.05). In contrast, there were no significant differences in the inferior mGCC at the chronic stage between the patients with NAION and those with ON (*t* = 2.547, *p* = 0.093).

**Conclusion:**

Various structural and microvascular changes were observed in patients with NAION and ON, indicating distinct features of the optic nerve during the different stages of NAION and ON. Peripapillary vascular density, measured using spectral domain OCT (SD-OCT), may be a biomarker to distinguish NAION from ON.

## 1 Introduction

Non-arteritic anterior ischemic optic neuropathy (NAION) is the most common cause of optic disc edema and optic neuropathy in adults aged >50 years (1). Some patients with NAION present with severe vision loss and corresponding unreliable visual fields at disease onset, while some patients with acute demyelinating optic neuritis (DON) mimic ischemic optic neuropathy, leading to potential misdiagnosis (2). Differentiating atypical NAION from acute DON is crucial because they have different prognoses and treatments.

Generally, NAION is distinguished from DON based on visual acuity, typical visual field, pupil, and color vision findings (3); however, confirming the diagnosis in the early stages of NAION, when the visual field manifestations with severe visual loss are very similar to those of DON, is challenging. In the chronic stage of NAION, an atrophied optic disc secondary to these two diseases makes it difficult to diagnose the disease.

Optical coherence tomography angiography (OCTA) has emerged as a non-invasive technique for imaging the retinal microvasculature. Previous studies on optical coherence tomography (OCT) have shown that optic neuritis (ON) causes more severe neuronal damage and an obvious decrease in the retinal nerve fiber layer (RNFL). Recently, the latest OCT and OCTA data on multiple sclerosis (MS) have been used to provide information on the possibility of using OCT/OCTA parameters as biomarkers for the screening, diagnosis, and monitoring of MS and neuromyelitis optical spectrum disorder (NMOSD) (4, 5).

In a previous study (6, 7), we created a change map of peripapillary vessel density in patients with NAION and found that the vessel density of the optic disc in the inferonasal and inferotemporal quadrants in patients with NAION did not decrease significantly over time. Therefore, OCT/OCTA can quantify changes in retinal structural thickness and microvascular density and may be a reliable tool to differentiate atypical NAION from ON.

In this observational cohort study, we utilized spectral domain OCT (SD-OCT) and swept-source OCTA (SS-OCTA) to record the retinal structural and microvascular changes in patients with NAION and DON compared with healthy individuals. We also aimed to determine the differences between OCT/OCTA parameters during acute and chronic stages and discover a biomarker to differentiate NAION from ON.

## 2 Methods

### 2.1 Inclusion and exclusion criteria

The Second Affiliated Hospital of Zhejiang University School of Medicine, Hangzhou, China, approved this prospective observational study. Written consent was obtained from all patients before their participation. A total of 27 healthy individuals, 23 patients with NAION, and 22 patients with DON, diagnosed at the Eye Center of the Second Affiliated Hospital of Zhejiang University School of Medicine between January 2023 and October 2023, were included in the study.

The inclusion criteria for patients with NAION were as follows: (1) sudden and painless loss of vision, (2) an optic disc-related defect in the visual field, (3) papillary edema following disease onset, (4) ocular and systemic risk factors related to NAION, (5) a relative afferent papillary defect (+) or aberrant visual-evoked potential (VEP), (6) normal C-reactive protein levels in routine blood examination, and (7) exclusion of other optic nerve diseases. The exclusion criteria for patients with NAION were as follows: (1) a refractive error greater than 3.0 D of astigmatism or 6.0 diopters (D) of spherical equivalent; (2) a history of intraocular surgery or laser treatment (excluding cataract surgery); (3) a history of retinal, optic, visual pathway, or central nerve systemic diseases; (4) poor cooperation with fixation or OCTA examination; and (5) opacity of the optic structure leading to inaccurate OCTA data (<7).

DON was diagnosed based on the Optic Neuritis Treatment Trial (8) and published guidelines (9). The inclusion criteria for patients with DON were as follows: (1) acute vision loss with or without pain from eyeball movement; (2) at least two of the following abnormalities: an abnormal pupil, a defect in the visual field, VEP abnormality, and color vision disturbance; (3) absence of ischemic and traumatic causes, compression, infiltration, and toxicity of the optic nerve, as well as nutritional, metabolic, or hereditary optic neuropathy; and (4) no history of glaucoma, retinal, or choroidal pathology. The exclusion criteria for patients with DON were as follows: (1) a refractive error greater than 3.0 D of astigmatism or 6.0 diopters (D) of spherical equivalent, (2) a history of intraocular surgery or laser treatment (excluding cataract surgery), (3) age less than 18 or over 80 years, (4) poor cooperation with fixation or OCTA examination, and (5) opacity of the optic structure leading to inaccurate OCTA data (<7).

For comparison, 27 healthy controls with no history of neurological or neuropsychological disease were included in our study. Participants with hypertension or diabetes and a history of ocular surgery, glaucoma, or other ophthalmologic diseases were also excluded.

All participants underwent medical optometry using the TOPCON automatic comprehensive optometry system, as well as intraocular pressure measurement, slit-lamp microscopy, fundus examinations, fundus photography, visual field examination (Octopus 900 24–2), VEP examination, OCT, and OCTA. The best-corrected visual acuity (BCVA) was assessed using a standard international far-visual chart and converted to logMAR notation. Fundus fluorescein angiography and VEP examination were performed when the patient was in good general physical condition.

### 2.2 OCTA and S domain OCT

In our study, we obtained OCT/OCTA data using the OSCAR-IB quality criteria (10) and APOSTEL recommendations (11).

**Figure 1:**
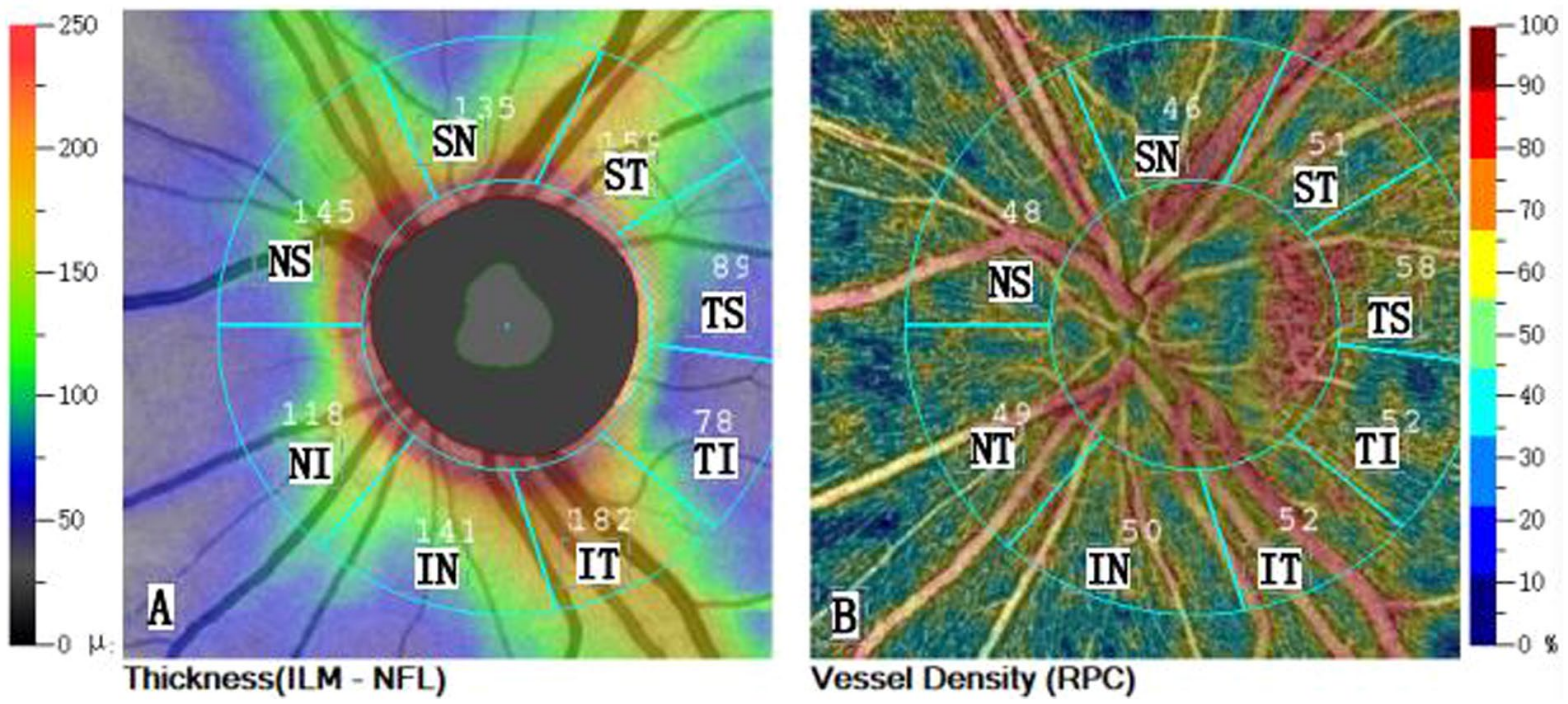
(A) The RNFL thickness map (ILM - NFL) of the left eye is based on the 2-4 mm diameter of the Garway-Heath zoning. Cool colors indicate areas of low thickness. Warm colors indicate areas of high thickness, Thickness values are entered in the partition. (B) The sectorial division analysis of the optical coherence tomography angiography (OCTA) image of the left eye. The OCTA en face image of the peripapillary area (2–4 mm diameter Garway-Heath partition) is automatically segmented in the radial peripapillary capillary layer. Cool colors indicate areas of low density. Warm colors indicate areas of high density. Blood flow density parameter values are filled in the partition. NS, nasosuperior; NI, nasoinferior; IN, inferonasal; IT, inferotemporal; TI, temporoinferior; TS, temporosuperior; ST, superotemporal; SN, superonasal; S, superior; I, inferior; N, nasal; T, temporal.

OCTA and spectral-domain OCT were performed using the AngioVue Imaging System (Optovue, Inc., Fremont, CA, United States; RTVue XR version 2018.1.0.43). We excluded images with a signal strength index of <7 and used the AngioVue system with 4.5× 4.5 mm automatic optic disc-centered peripapillary scans. Whole en face image vessel density (wiVD) and intradisc vessel density (idVD) were calculated using the 4.5 × 4.5 mm cube scan. We also collected data on the superficial VD of the radial peripapillary capillaries (RPCs) and the thickness of the RNFL from an area with diameters between 2 and 4 mm (Figure 1). This region was divided into eight sectors: [temporosuperior (TS), superotemporal (ST), superonasal (SN), nasosuperior (NS), nasoinferior (NI), inferonasal (IN), temporoinferior (TI), and inferotemporal (IT)].

We used a 6 × 6 mm foveal-centered macular cube scan to measure the thickness of the ganglion cell complex (GCC), which extends from the inner limit membrane to the inner plexiform layer (IPL). This region comprises the RNFL, ganglion cell layer (GCL), and IPL. Subsequently, we recorded the average, superior, and inferior hemispheric GCC thicknesses in the macular and parafoveal areas.

The retinal layers were automatically segmented to visualize the superficial vascular plexus (SVP) in a slab from the internal limiting membrane to 10 μm above the IPL, as well as the deep vascular plexus from 10 μm above the IPL to 10 μm beneath the outer plexiform layer.

### 2.3 Statistical analysis

The Shapiro–Wilk test was used to evaluate data distribution. Normally distributed quantitative data were expressed as the mean ± standard deviation (SD), while categorical data were reported as percentages (%). Non-normally distributed quantitative data were expressed as medians (interquartile range: P25, P75) and compared using the Mann–Whitney U test. A matched *t*-test for the categorical data was used to compare and analyze the differences in each group during different periods, and the Student–Newman–Keuls (S–N–K) method was used to compare the two groups. All data analyses were performed using SPSS statistical software version 26 (SPSS Inc., Chicago, Illinois, United States). Statistical significance was set at a *p*-value of <0.05.

## 3 Results

### 3.1 Study population

A total of 23 patients with NAION, 22 patients with DON, and 27 healthy controls were enrolled in this study. In this cohort, no significant differences were observed between the patients and controls regarding age, sex, laterality of eyes, or follow-up time. The BCVA of the patients with NAION changed from 0.57 ± 0.43 to 0.51 ± 0.44, while that of the patients with DON changed from 1.53 ± 1.09 to 0.81 ± 1.04 with time. In comparison with controls, both groups showed a decrease in BCVA during the acute stage (*p* < 0.001) and the chronic stage (*p* < 0.034); however, there was no difference in MD and BCVA at the chronic stage between the groups (*p* < 0.001), and no significant improvement was observed in the initial MD and BCVA for patients with NAION (*p* = 0.524, 0.435) and DON (*p* = 0.922, 0.073). The mean follow-up time was 202.83 ± 16.79 days for the patients with NAION and 93.75 ± 10.61 days for the patients with DON.

### 3.2 Differences in RPC vessel densities among the patients with NAION and DON and the controls

**Figure 2:**
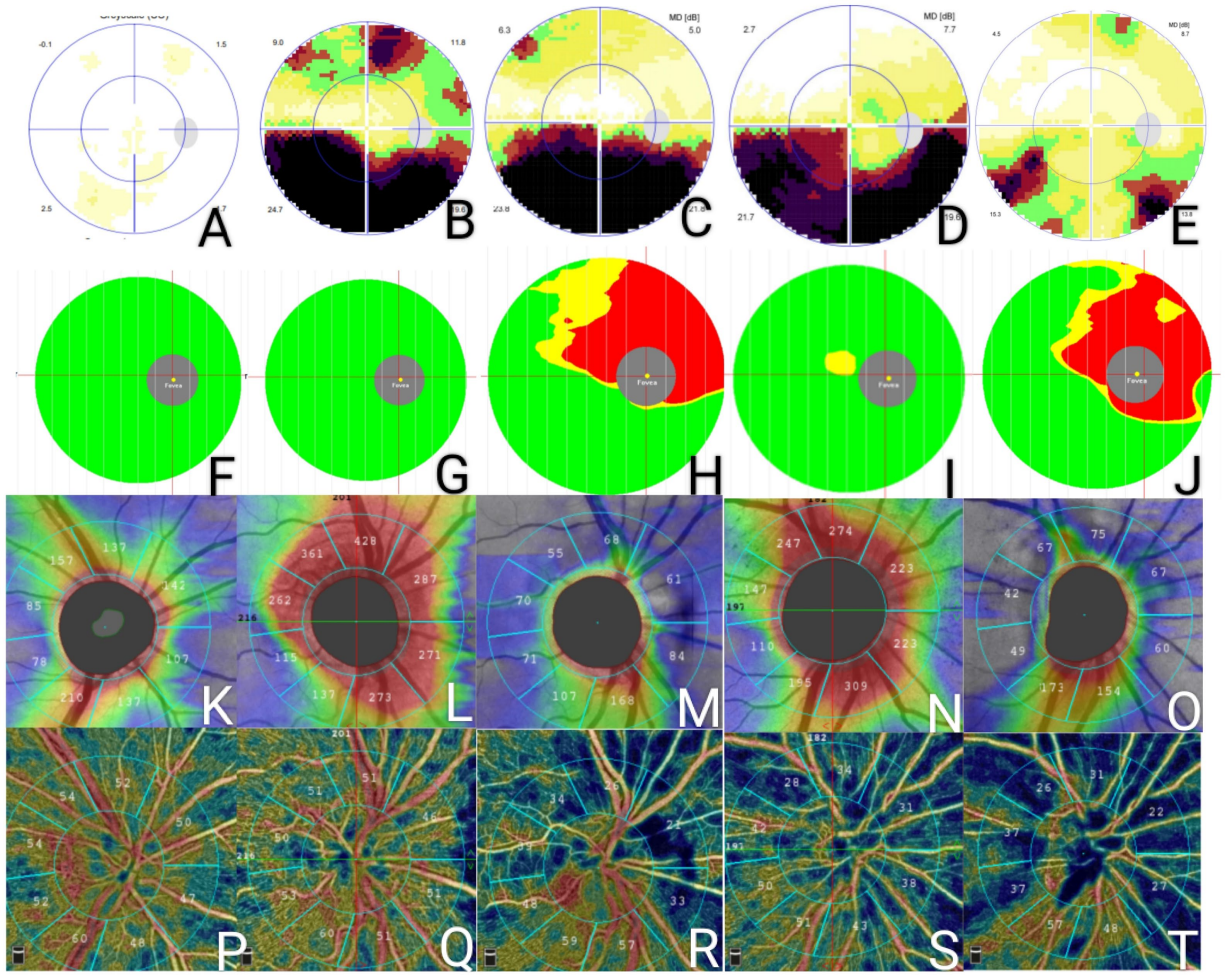
A 50-year-old female patient with a normal visual field (A), mGCC measurements (F), pRNFL (K), and RPC (P). A 55-year-old female patient with NAION in her right eye demonstrated improvement in her visual field from the initial (B) to the chronic stage (C); however, the mGCC measurements (G,H), pRNFL (L,M), and RPC (Q,R) all decreased from the onset of the disease to the chronic stage. A 39-year-old female patient with DON in her right eye demonstrated improvement in her visual field after 3 months (D,E), while the mGCC measurements (I,J), pRNFL (N,O), and RPC (S,T) all decreased over time. mGCC, macular ganglion cells; pRNFL, peripapillary retinal nerve fiber layer; RPC, radial papillary capillaries; NAION, non-arteritic anterior ischemic optic neuropathy; DON, demyelinating optic neuritis.

All areas of the RPCs (except for the inferotemporal RPC in the NAION group) in the patients with NAION and DON decreased from the acute to the chronic stage (*p* < 0.05); the inferotemporal RPC in the patients with NAION did not decrease with time (*p* = 0.117). There were no significant differences in any areas of the RPCs from the acute to the chronic stage in the controls. Among the three groups, there were no significant differences in the nasoinferior and temporoinferior RPC at the acute stage (*p* = 0.236, *p* = 0.767) and no difference in the inferonasal and inferotemporal RPC at both stages (*p* = 0.154, 0.078; *p* = 0.701, 0.119) (Table 1). We observed typical changes in a 55-year-old female patient with NAION and a 39-year-old female patient with DON (Figure 2). Compared with the normal control (a 50-year-old female patient), one patient exhibited a progressive decrease in the superior RPC, whole peripapillary RNFL, and superior macular ganglion cell complex (mGCC), while the other patient showed a reduction in the entire area of the RPCs, peripapillary RNFL, and mGCC (Figure 2).

**Table 1:** The differences in RPC vessel densities in patients with NAION/DON and controls at different stages.

Index	NAION	DON	Controls	*F*	*P*
RPC1-AVERAGE, %	45.47 ± 3.46	43.91 ± 4.84	51.91 ± 1.68	10.992	<0.001
RPC2-AVERAGE, %	36.81 ± 6.09	34.49 ± 6.79	51.10 ± 1.63	19.604	<0.001
*t*	6.619	5.392	11.038		
*P*	<0.001	<0.001	<0.001		
RPC1-TS, %	47.05 ± 6.59	46.13 ± 6.90	55.00 ± 3.11	5.048	0.012
RPC2-TS, %	40.83 ± 9.35	37.71 ± 4.89	55.57 ± 2.94	11.308	<0.001
*t*	3.036	4.151	−0.934		
*P*	0.006	<0.001	0.386		
*RPC1-ST*, %	39.70 ± 4.85	38.88 ± 9.52	53.86 ± 1.77	17.696	<0.001
*RPC2-ST*, %	26.70 ± 6.55	30.29 ± 12.88	53.29 ± 2.56	32.916	<0.001
*t*	7.566	3.215	1.082		
*P*	<0.001	<0.001	0.321		
*RPC1-SN*, %	38.90 ± 5.21	40.50 ± 5.90	51.00 ± 4.32	14.317	<0.001
*RPC2-SN*, %	25.39 ± 6.34	28.86 ± 8.23	50.43 ± 4.20	41.297	<0.001
*t*	8.945	4.887	0.603		
*P*	<0.001	<0.001	0.569		
*RPC1-NS*, %	41.81 ± 6.07	42.38 ± 6.46	49.71 ± 3.09	5.170	0.011
*RPC2-NS*, %	30.43 ± 8.78	28.43 ± 6.73	49.00 ± 3.92	17.151	<0.001
*t*	6.625	4.625	1.109		
*P*	<0.001	<0.001	0.310		
*RPC1-NI*, %	44.82 ± 6.30	43.50 ± 4.99	48.43 ± 4.31	1.508	0.236
*RPC2-NI*, %	34.70 ± 8.83	33.29 ± 7.11	48.57 ± 4.31	9.225	0.001
*t*	5.563	4.527	−0.311		
*P*	<0.001	<0.004	0.766		
*RPC1-IN*, %	48.43 ± 4.58	44.62 ± 6.23	49.14 ± 5.05	1.982	0.154
*RPC2-IN*, %	43.39 ± 10.5	37.29 ± 9.96	49.43 ± 5.26	2.753	0.078
*t*	2.508	2.721	−0.795		
*P*	0.021	<0.001	0.457		
*RPC1-IT*, %	53.52 ± 5.93	53.13 ± 17.22	56.71 ± 3.86	0.359	0.701
*RPC2-IT*, %	48.96 ± 13.37	41.57 ± 14.35	55.71 ± 3.86	2.265	0.119
*t*	1.639	2.554	0.823		
*P*	0.117	0.043	0.442		
*RPC1-TI*, %	51.36 ± 6.72	51.75 ± 16.28	54.29 ± 4.57	0.267	0.767
*RPC2-TI*, %	45.17 ± 9.18	33.86 ± 6.15	53.86 ± 4.38	10.95	<0.001
*t*	4.169	3.037	1.441		
*P*	<0.001	0.023	0.200		

RPC, radial papillary capillaries; NAION, nonarteritic anterior ischemic optic neuropathy; DON, demyelinating optic neuritis; TS, temporosuperior; ST, superotemporal; SN, superonasal; NS, nasosuperior; NI, nasoinferior; IN, inferonasal; IT, inferotemporal; TI, temporoinferior. 1 stands for the data from the acute stage (7–21 days) 2 stands for the data from the chronic stage (> 90 days).

### 3.3 Differences in the peripapillary RNFL among the patients with NAION and DON and the controls

Peripapillary RNFL thickness decreased from the acute to the chronic stage in the patients with NAION and DON (*p* < 0.05). Compared with the controls, all optic nerve structures in the patients decreased or became thinner over time, with no differences in the inferotemporal and temporoinferior regions of the peripapillary RNFL at either stage (*p* = 0.087, 0.104; *p* = 0.175, 0.131) and no differences in the inferonasal region at the chronic stage among the three groups (*p* = 0.45) (Table 2).

**Table 2:** The differences of pRNFL in patients with NAION, DON and controls at different stages.

Index	NAION	DON	Controls	*F*	*P*
RNFL1-AVERAGE, μm	237.19 ± 56.85	222.00 ± 60.04	125.14 ± 7.01	12.250	<0.001
RNFL2-AVERAGE, μm	83.57 ± 26.16	77.71 ± 12.70	125.43 ± 6.70	11.257	<0.001
*t*	10.837	6.027	−1.000		
*P*	<0.001	<0.001	0.356		
RNFL1-TS, μm	176.95 ± 111.91	107.38 ± 26.03	86.29 ± 7.89	3.672	0.036
RNFL2-TS, μm	58.91 ± 17.94	45.57 ± 8.32	87.57 ± 9.31	14.17	<0.001
*t*	4.996	5.102	−1.536		
*P*	<0.001	<0.001	0.175		
RNFL1-ST, μm	267.13 ± 97.50	212.63 ± 68.30	149.43 ± 13.79	5.689	0.007
RNFL2-ST, μm	55.96 ± 13.97	72.29 ± 28.58	149.57 ± 13.93	77.474	<0.001
*t*	10.945	5.017	−0.420		
*P*	<0.001	<0.001	0.689		
*RNFL1-SN*, μm	283.33 ± 113.27	271.38 ± 116.09	145.43 ± 23.78	4.796	0.015
*RNFL2-SN*, μm	61.61 ± 19.57	72.57 ± 14.30	145.43 ± 23.34	50.176	<0.001
*t*	9.908	4.451	<0.001		
*P*	<0.001	<0.001	1.000		
*RNFL1-NS*, μm	232.62 ± 77.82	226.88 ± 80.22	123.00 ± 15.73	6.496	0.004
*RNFL2-NS*, μm	64.39 ± 17.83	73.00 ± 20.36	124.29 ± 16.12	29.889	<0.001
*t*	10.347	4.424	−1.441		
*P*	<0.001	<0.001	0.200		
*RNFL1-NI*, μm	217.27 ± 90.71	220.87 ± 106.60	97.57 ± 11.27	5.556	0.008
*RNFL2-NI*, μm	76.13 ± 19.82	68.29 ± 13.17	99.86 ± 13.50	6.369	0.004
*t*	7.235	3.612	−1.416		
*P*	<0.001	0.011	0.207		
*RNFL1-IN*, μm	302.43 ± 95.66	326.75 ± 128.57	145.00 ± 13.14	8.550	0.001
*RNFL2-IN*, μm	132.35 ± 74.32	104.43 ± 36.20	145.00 ± 13.09	0.817	0.450
*t*	5.967	4.618	<0.001		
*P*	<0.001	0.004	1.000		
*RNFL1-IT*, μm	282.00 ± 116.29	277.88 ± 101.21	182.29 ± 18.87	2.630	0.087
*RNFL2-IT*, μm	149.74 ± 86.05	94.86 ± 51.90	178.00 ± 14.90	2.426	0.104
*t*	3.696	3.655	1.082		
*P*	0.001	0.011	0.321		
*RNFL1-TI*, μm	158.00 ± 106.93	133.00 ± 65.47	84.29 ± 10.18	1.832	0.175
*RNFL2-TI*, μm	79.17 ± 44.90	50.00 ± 7.83	87.00 ± 11.37	2.161	0.131
*t*	3.014	3.416	−0.887		
*P*	0.007	0.014	0.409		

pRNFL, peripapillary retinal nerve fiber layer; NAION, nonarteritic anterior ischemic optic neuropathy; DON, demyelinating optic neuritis; TS, temporosuperior; ST, superotemporal; SN, superonasal; NS, nasosuperior; NI, nasoinferior; IN, inferonasal; IT, inferotemporal; TI, temporoinferior. 1 stands for the data from the acute stage (7–21 days), 2 stands for the data from the chronic stage (>90 days).

### 3.4 Differences in mGCC thickness among the patients with NAION and DON and the controls

Compared with the controls, the average thickness and superior mGCC decreased from the acute to the chronic stage in the patients with NAION (*p* < 0.001, *p* < 0.001) and DON (*p* = 0.003, *p* = 0.002). The thickness of the inferior mGCC decreased from the acute to the chronic stage in the patients with DON (*p* = 0.009) but did not decrease in the patients with NAION (*p* = 0.413); it remained unchanged in both stages in the control group (*p* = 0.867, *p* = 0.093). These differences are listed in Table 3.

**Table 3:** The changes of mGCC in patients with NAION, DON and controls at different stages.

Index	NAION	DON	Controls	*F*	*P*
GCC1-AVERAGE, μm	98.26 ± 8.8	90.38 ± 9.36	100.14 ± 3.02	3.368	0.046
GCC2-AVERAGE, μm	83.22 ± 11.66	70.57 ± 10.5	100 ± 3	14.077	<0.001
*t*	5.558	4.87	1.000		
*P*	<0.001	0.003	0.356		
S-mGCC1, μm	92.7 ± 12.51	84.75 ± 9.65	99.86 ± 3.53	3.607	0.038
S-mGCC2, μm	71.87 ± 10.44	68 ± 7.16	99.43 ± 3.41	28.666	<0.001
*t*	7.395	5.49	1.000		
*P*	<0.001	0.002	0.356		
I-mGCC1, μm	99.47 ± 23.75	95.88 ± 12.01	101 ± 4.16	0.144	0.867
I-mGCC2, μm	94.39 ± 16.49	73.43 ± 14.71	86.44 ± 38.33	2.547	0.093
*t*	0.834	3.771	1.012		
*P*	0.413	0.009	0.351		

mGCC, macular ganglion cells; S-mGCC, superior mGCC; I-mGCC, inferior mGCC; NAION, nonarteritic anterior ischemic optic neuropathy; DON, demyelinating optic neuritis. 1 stands for the data from the acute stage (7–21 days) 2 stands for the data from the chronic stage (>90 days).

## 4 Discussion

In this study, we performed longitudinal measurements of RPC and mGCC thicknesses and peripapillary RNFL thickness in the acute and chronic phases of NAION and DON. We compared the results with those of the controls.

Recent studies utilizing OCTA have reported a significant reduction in the macular SVP in patients with NMOSD and myelin oligodendrocyte glycoprotein antibody-associated disease (MOGAD). They found thinner ganglion cell IPL and reduced macular SVP density in patients with MOGAD compared to patients with NMOSD and those with a history of ON (12, 13). Moreover, ON attacks were more recurrent in patients with MOGAD; therefore, the changes observed by OCT and OCTA may reflect severe neurodegeneration and microvascular impairment. Vessel densities in the peripapillary and macular areas are reduced in patients with NMOSD without a previous episode of ON (14). Fu et al. revealed subclinical loss of the RNFL and ganglion cell inner plexiform layer (GCIPL) in NMOSD eyes using OCT, even in eyes with no history of ON (15). This indicates that damage to the body and axons of retinal ganglion cells (RGCs) is more severe in NMOSD eyes following an episode of ON. In our study, the eyes with DON had thinner peripapillary RNFL and mGCC thicknesses in all areas compared with the controls, while the eyes with NAION had thinner pRNFL thickness in all areas and thicker thickness in the inferior mGCC. We speculate that acute ischemia causes irreversible impairment to the ganglion cell layer (GCL) in the superior area of the optic disc, suggesting that the thinning of the mGCC is secondary to peripapillary axonal degeneration in NAION.

Rogaczewska et al. demonstrated that RPC vessel density is more significantly reduced in patients with NMOSD compared to those with MS, indicating that the pathological mechanisms differ between the two groups. They considered the preference for the superior and inferior sectors to be useful as differential diagnostic markers for these two diseases (16). In our study, while several patients exhibited similar visual field defects and similar mGCC reduction, the degree of RPC reduction in the eyes with DON was found to be diffused and irregular, whereas the degree of RPC reduction in the eyes with NAION was lighter around the superonasal and superotemporal areas. The RPC reduction resulted from the accompanying retinal ganglion cell loss and metabolic demand, which is a common outcome of optic neuropathies. We could not determine the exact cause of the diseases. Therefore, we suggest that OCTA can be used to reveal differences and evaluate the severity of damage.

Relatively, more studies have been conducted on the RPCs in patients with NAION. Liu et al. reported a more drastic reduction in the superior RPC, suggesting a sectoral reduction in the RPC as the optic disc edema subsided, along with a decrease in superficial vascular density and unchanged macular deep vascular density (17). In addition, Augstburger et al. reported normal deep vascular complex, decreased superficial complex plexus during the initial stage (1 month after the presentation for NAION), and decreased whole en face image RPC within 6 months (18). We observed a more specific tendency of the RPCs in the patients with NAION (6). The RPCs decrease from the acute to the chronic stage and remain stable at week 12 (7). In our study, the eyes with NAION had thinner peripapillary RNFL thickness in all areas and a thicker inferior mGCC than the eyes with DON. We speculate that acute ischemia results in irreversible GCL impairment in the superior area of the optic disc, suggesting that mGCC thinning is secondary to peripapillary axonal degeneration in NAION. We posit that the tendency of RPC reduction around the ischemic area over time is consistent with the pathological mechanism of NAION.

Few studies have explored the differences in the RPCs between patients with DON and those with NAION. Fard et al. compared the pale optic disc in both groups of patients using OCTA and found that peripapillary vessel density measurements and RNFL thickness did not distinguish between previous episodes of ON and those of NAION (19). Unlike the results of their study, we found that the inferotemporal RPC decreased from the acute to the chronic stage in the patients with DON but not in those with NAION. Nevertheless, we posit that RPC reduction reflects the decreased need for blood flow in the RNFL and GCL, regardless of the cause.

A comparison of the differences in the optic disc in the acute and chronic stages revealed the inferotemporal RPC as a unique characteristic in the patients with NAION but not in those with DON. We also found that most patients with NAION showed decreased inferior mGCC compared to the patients with DON. This difference in the retinal structure may be linked to the pathology of NAION as the superior optic nerve is mostly located in the ischemic area, and diffused atrophy is caused by optic inflammation and neural degeneration in patients with DON.

Similar to some studies on MS (20), the thickness of both the peripapillary RNFL and mGCC decreased in our patients with DON, suggesting that these measurements reflect the severity of the disease. Moreover, both neuronal loss and axonal degeneration are present in this disease. We can use GCL thinning to access the entire central nervous system (21, 22). We could not further classify optic neuritis because of the small number of cases. We cannot differentiate neuronal loss and axonal degeneration in DON and MS; therefore, it is particularly important to observe changes at different stages after the onset of inflammation. However, OCTA can help us speculate the ischemic area based on our study.

Based on current studies that compare DON and MS with NAION using OCTA (20, 23), we observed a thinner average RNFL thickness and a lower vessel density of the average RPC in patients with DON in our study, indicating that damage is more severe in patients with DON compared to those with MS and DON. It is possible that our country has fewer patients with MS, as the patients in our study mainly suffered from DON without MS. In contrast to limited studies investigating patients with MS, our study provides a more comprehensive and specific analysis of peripapillary RPC loss in patients with ON compared to those with NAION.

Ultimately, the outcome of both diseases is a decrease in the RNFL, mGCC, and RPC. Nevertheless, we observed not only localized changes in patients with NAION but also diffuse changes in patients with ON by comparing data from eight partitions across the acute to chronic stages for the first time. More specific and earlier findings are expected to provide biological markers for early differential diagnosis.

Our study had some limitations that should be acknowledged. First, this pilot study had a small sample size in our hospital cohort, which may limit the generalizability of the findings to the general population. Second, there may be a selection bias owing to the exclusion of individuals from the groups. Third, although we excluded patients in the first week, the potential impact of optic nerve head swelling at the acute stage of blood flow signal detection cannot be dismissed. Fourth, to assess the serial changes in retinal vessel density in patients with NAION and DON, we collected longitudinal data over time from the same patients from the onset of the diseases to the chronic atrophic stage. Finally, we did not record superficial or deep macrovascular changes in the macula or choroid. Therefore, further studies with larger patient populations and specific disease stages are warranted.

## 5 Conclusion

Our study demonstrated distinct structural and microvascular changes between the patients with NAION and those with DON, suggesting different mechanisms of optic nerve damage between the two conditions. Compared to the patients with NAION and the controls, the inferotemporal RPC and inferior mGCC in the patients with DON decreased significantly. In contrast, the other quadrants of the RPCs (except for the inferotemporal region), the superior mGCC, and the whole area of the RNFL in the patients with NAION and DON decreased compared with the healthy individuals. The inferotemporal RPC, as assessed using OCTA, can be used as a biomarker to distinguish NAION from DON. Furthermore, OCT/OCTA can aid in distinguishing atypical NAION from DON in cases with overlapping clinical features.

## Data Availability

The original contributions presented in the study are included in the article/supplementary material, further inquiries can be directed to the corresponding author.
